# Environmental Light and Its Relationship with Electromagnetic Resonances of Biomolecular Interactions, as Predicted by the Resonant Recognition Model

**DOI:** 10.3390/ijerph13070647

**Published:** 2016-06-29

**Authors:** Irena Cosic, Drasko Cosic, Katarina Lazar

**Affiliations:** 1 College of Science, Engineering and Health, RMIT University, La Trobe Street, Melbourne, Victoria 3000, Australia; 2 AMALNA Consulting, 46 Second St., Black Rock, Victoria 3193, Australia; draskocosic@yahoo.com.au (D.C.); katcosic@yahoo.com.au (K.L.)

**Keywords:** environmental light, protein and DNA interactions, sun light, artificial light, protein, DNA, biomolecular electromagnetism, resonant recognition model

## Abstract

The meaning and influence of light to biomolecular interactions, and consequently to health, has been analyzed using the Resonant Recognition Model (RRM). The RRM proposes that biological processes/interactions are based on electromagnetic resonances between interacting biomolecules at specific electromagnetic frequencies within the infra-red, visible and ultra-violet frequency ranges, where each interaction can be identified by the certain frequency critical for resonant activation of specific biological activities of proteins and DNA. We found that: (1) the various biological interactions could be grouped according to their resonant frequency into super families of these functions, enabling simpler analyses of these interactions and consequently analyses of influence of electromagnetic frequencies to health; (2) the RRM spectrum of all analyzed biological functions/interactions is the same as the spectrum of the sun light on the Earth, which is in accordance with fact that life is sustained by the sun light; (3) the water is transparent to RRM frequencies, enabling proteins and DNA to interact without loss of energy; (4) the spectrum of some artificial sources of light, as opposed to the sun light, do not cover the whole RRM spectrum, causing concerns for disturbance to some biological functions and consequently we speculate that it can influence health.

## 1. Introduction

It is well known that the life on Earth has been sustained by the electromagnetic energy from sunlight. In primitive organisms and plants the sunlight directly influences biological processes, while, in more complex organisms, it has a more indirect role. In these organisms, due to their more complex structures, sunlight cannot penetrate into each cell, therefore, they have to create their own “internal sun” energy to drive the selectivity of biological processes in their cells, in the same manner as they were initially sustained by the sun [1,2].

The selectivity (specificity) of biological processes is driven by the information contained within linear macromolecules: DNA and proteins. While information in DNA is written within the long sequences using different combinations of four different nucleotides, information in proteins is also written within long sequences, but using different combinations of 20 amino acids. While DNA carries the complete backup information of any organism, proteins are macromolecules that read the necessary parts of DNA information to actually perform all selective biological activity through a number of very specific interactions. The Resonant Recognition Model (RRM) model proposes that macromolecular selective interactions are based on electromagnetic resonant energy transfer between macromolecules in the range of infra-red, visible and ultra-violet light, and, thus, could mimic the specificity enabled by different frequencies (wavelengths) of sunlight [3,4]. By applying RRM, it is possible to identify and calculate relevant frequencies, critical for resonant activation of specific biological activities of proteins and DNA [5,6,7,8,9,10,11].

Here, we discuss:

- whole RRM spectrum for different biological functions of proteins and DNA;
- grouping of different biological functions into super families;
- comparison of RRM spectrum with the water absorption spectrum, spectrum of sunlight and spectrum of some artificial sources of light.

## 2. Materials and Methods

### Resonant Recognition Model

RRM is based on the findings that certain periodicities within the distribution of energy of delocalized electrons along a protein (DNA/RNA) molecule are critical for protein (DNA/RNA) biological function and/or interaction with their targets [3,4,12]. If charge transfer through these macromolecules is introduced, then the charge moving through the macromolecular backbone can produce electromagnetic radiation, absorption and resonance with spectral characteristics corresponding to the energy distribution [3,4,5,6,7,8].

RRM enables the calculation of these spectral characteristics, by assigning each amino acid a physical parameter representing the energy of delocalized electrons of each amino acid. Comparing Fourier spectra for these energy distributions, using cross-spectral function, it has been found that proteins sharing the same biological function/interaction share the same periodicity (frequency) within the energy distribution along the macromolecule [3,4]. Furthermore, it has been shown that interacting proteins and their targets share the same characteristic frequency, but have an opposite phase at a characteristic frequency [3,4,12]. Thus, it has been proposed that the RRM frequencies characterize, not only a general function, but also recognition and interaction between the particular macromolecule and its target, which can then be considered to be resonant recognition. This could be achieved with resonant energy transfer between the interacting macromolecules through oscillations of a physical field, which is electromagnetic in nature. Since there is evidence that proteins and DNA have certain conducting or semi-conducting properties, a charge moving through the macromolecular backbone and passing different energy stages, caused by different amino acid or nucleotide side groups, can produce sufficient conditions for a specific electromagnetic radiation or absorption. The frequency ranges of this field depend on the charge velocity. RRM proposes that the charge is travelling through the macromolecular backbone at an estimated velocity of 7.87 × 10^5^ m/s [3,4]. For this velocity, and with the distance between amino acids in a protein molecule of 3.8 Å, the frequency of protein interactions was estimated to be in the range of 10^13^ Hz and 10^15^ Hz. Therefore, the estimated frequency range for both amino acid and nucleotide macromolecules includes infra-red, visible and ultra-violet light. To support this idea, we compared our computational predictions with a number of published experimental results [3,4,8]:

- Laser light growth promotion of cells, by using the particular frequencies of light to produce the similar effect to that of growth factor proteins;
- chymotrypsin activation (increase of enzyme activity) achieved by laser light radiation in a range of 850–860 nm;
- activation of highly homologous plant photoreceptors which, although being very homologous, absorb different wavelengths of light;
- photo activated proteins, e.g., rhodopsin, flavodoxin, etc.

These comparisons have shown a strong linear correlation between frequencies, as calculated using the RRM method and experimentally measured characteristic frequencies, with a slope factor of K = 201 [3,4,8]. This finding is in parallel with the frequency range previously associated with the RRM numerical frequency spectrum, which has been calculated from the charge velocities through the protein backbone. This correlation can be represented as following:

λ = K/frrm
(1)

where λ is the wavelength of light irradiation in nm, which can influence a particular biological process, frrm is a RRM numerical frequency, and K is coefficient of this linear correlation.

We applied this concept to a number of proteins and DNA examples [3,4,5,6,7,8]. The concept has been also experimentally tested by predicting the electromagnetic frequencies for l-Lactate Dehydrogenase [8], whereby radiating l-Lactate Dehydrogenase with predicted calculated electromagnetic frequencies achieved a significant change in enzyme activity. The concept has also been tested independently on experimental measurements of photon emission from dying melanoma cells [9], on photon emission from lethal and non-lethal Ebola strains [10], as well as on the classic signaling pathway, JAK-STAT, traditionally composed of nine sequential protein interactions [11].

Keeping all this in mind, we propose that the RRM concept is an excellent predictor for the selective interactions, biological processes and pathways of proteins and DNA in living cells. In our previous work, we have calculated a large number of specific frequencies for different protein and DNA biological functions and interactions. These frequencies, and related functions, are presented in Table 1.

## 3. Results

We applied the RRM model to a large number of protein and DNA functional groups, and the identified characteristic RRM frequencies are presented in Table 1. The chosen protein and DNA sequences have been predominantly selected based on the availability of sequences, proven biological functions and existing experimental results. Therefore, there is a possibility that new functional groups and related RRM frequencies will appear in future research.

It can be observed from the calculated RRM frequencies, that there are interesting groupings of biological functions into functional super families. For example, it can be observed that protein and DNA functions that are related to the uncontrolled cell growth super family (like oncogenes, antitumor agents, TNFs, etc.), are all within the frequency range between 0.031 and 0.054, as highlighted in red, in Table 1. Similarly, the super family of viral and bacterial infections are grouped together and highlighted in orange, the super family related to controlled growth is highlighted in yellow, while the super family related to enzyme activity is highlighted in green, as presented in Table 1. It appears that there are two smaller super families: Structural proteins, highlighted in blue, and proteins related to blue light absorption/emission, highlighted in violet. In addition, there are other functional groups that cannot be grouped into the super families at this point in time, but, with more knowledge on protein and DNA sequences and their functions, there are possibilities for more super families to be identified.

**Figure 1:**
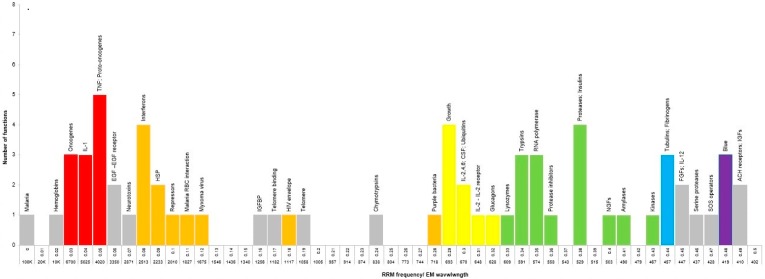
Number of functional groups within each Resonant Recognition Model (RRM) frequency range of 0.01. *X* axis represent RRM frequency in steps of 0.01, as well as corresponding electromagnetic frequency in nm. *Y* axis represent number of functional groups. Names of functional groups are noted above each bar. Super families are colored as in Table 1.

The results presented in Table 1 have been also presented graphically in Figure 1, where each RRM frequency range of 0.01 is presented with number of functions within that range. This graphical presentation enables a better visualization of functional groupings of protein and DNA macromolecules, based on RRM frequencies. All identified super families have been colored in accordance to the colors used in Table 1.

Based on the RRM principle, as described in Materials and Methods section, the numerical RRM frequencies represent oscillations of electromagnetic fields, which are relevant for specific biological functions/interactions. The frequencies of these electromagnetic oscillations are calculated in nm for each biological function, and are presented in Table 1, column 2. For each 0.1 of the RRM frequency range, as presented in Figure 1, the corresponding frequencies of electromagnetic oscillations have been calculated in nm and presented along the *X* axis in Figure 1.

## 4. Discussion

### 4.1. Functional Super Families

As presented above, the protein and DNA sequences can be grouped into functional super families, based on the calculated RRM frequencies. The most interesting result is that there are distinct RRM characteristics for uncontrolled and controlled cell growth, which presents an enormous opportunity for understanding cell transformation and cell growth control. Having such characteristics at the molecular level provides a new aspect to influence uncontrolled cell growth and consequently combat cancer formation and growth. Some preliminary results have experimentally shown that it is possible to use the RRM to design peptides that can interfere with oncogenic transformation [13,14]. RRM proposes that the characteristic of uncontrolled cell growth is in a specific range of electromagnetic radiation, which has been proved by experimental measurements with cancer tissue [9,15]. In addition, the design of bioactive peptides, using the RRM, have been experimentally tested on examples of cell growth control [16] and vaccine development [17], as well as electromagnetic radiation, as predicted by RRM, can interfere with infections such as malaria [18] and Ebola [10].

### 4.2. Water Absorption

**Figure 2:**
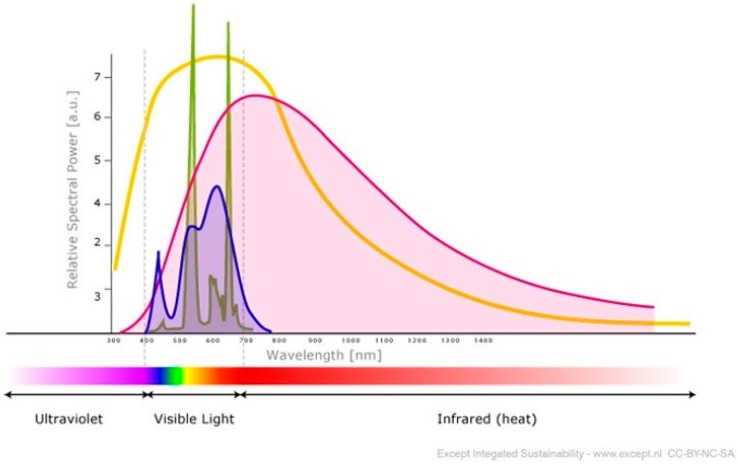
Diagram of the spectrum a LED lamp (blue), a CFL (green) and an incandescent (purple) superimposed the solar spectrum (yellow).

It can be observed that the whole spectrum of frequencies, as predicted by RRM to be relevant for the biological activity of proteins and DNA, also covering the same spectrum as the spectrum of sunlight on the Earth’s surface, as represented by the yellow line in Figure 2 [19]. This finding was as expected, since sunlight sustains all life processes on Earth. This implies that protein and DNA activity is mimicking the role of the sun within the biological functions of the cells.

It is also important to note that all biological processes in living cells occur in a water medium, which is only transparent for electromagnetic frequencies in the spectrum encompassing mostly visible light, just as predicted by RRM. This means the water medium enables electromagnetic radiation of these frequencies to be transferred between macromolecules without any loss of energy and, therefore, maximizing the efficiency of these interactions.

### 4.3. Artificial Light

The frequency range for biological functions has been found to be the same as the frequency range of sunlight on Earth, as described above. This reinforces the fact that life is sustained by the energy from sunlight. This also means that environmental sunlight is natural source of life on Earth. Humans, however, are spending more and more time under artificial lights, which may not have the same spectral characteristics as sunlight and, therefore, may induce a debalance in some biological functions. The spectrums of some artificial light sources have been presented in Figure 2 [19]. It is interesting to note that incandescent light, as represented by the purple line, has a similar spectrum shape as sunlight. In contrast, LED (light-emitting diode) light, as represented with the blue line, and CFL (compact fluorescent light) light, as represented by the green line, have distinct peaks at certain frequencies within the spectrum, while they are missing many of the other frequencies from the sunlight spectrum.

We have compared the spectrums of LED and CFL artificial lights with the frequencies of particular biological functions, as calculated by the RRM model. It can be observed that the artificial lights have strong radiation relevant to enzymes and that control growth activity, while they are missing frequencies related to tumor regulation and viral-bacterial infections. We speculate that these findings could suggest the possibility that, under such artificial lights, tumor regulation could be affected. In addition, we speculate that lack of light frequencies in the range of bacterial and viral infection control could lead to a higher susceptibility to these infections. Although, the majority of biological functions within the human organism are protected from electromagnetic radiation within the observed spectrum by skin and clothes, these artificial lights might still cause some distortions to biological functions in humans due to the lack of a full spectrum of sunlight. For example, there is experimental evidence that the specific photon energies of the weak magnetic field of the LED wavelength pulses are stored in malignant cells [20].

## 5. Conclusions

Here, for the first time, environmental light electromagnetic radiation was investigated as a source and influence on biomolecular interactions, related biological functions and consequent health effects. The relationship between our theoretical model, RRM, and sunlight’s ability to sustain of life, gives a possible explanation of how life processes may have evolved and are controlled in more complex organisms, where the sunlight cannot penetrate all cells and cellular processes. In addition, although biological processes are currently regarded as a large number of different events, we have shown that they are grouped in a relatively small number of general functions, enabling a simpler approach in understanding macromolecular interactions, biological functions and related health effects. In that light, the role of water and the possible influence of artificial light on biological processes have been shown. Keeping all this in mind, we can conclude that the Resonant Recognition Model (RRM) is a powerful tool in the analysis of protein and DNA functions/interactions, which are proposed to be based on resonant electromagnetic energy transfer.

## Figures and Tables

**Table 1:** Characteristic Resonant Recognition Model (RRM) frequencies for different biological functions of protein and DNA macromolecules. Column 1 represents the numerical RRM frequency. Column 2 represents the corresponding electromagnetic radiation in nm. Column 3 represents name of functional group of proteins and DNA. Column 4 represents super family of number of functional groups, which are also highlighted in different colors.

RRM Frequency	Nano Meters	Functional Group	Super Family
0.002	100 K	Circumsporosoite, PfEMP1, EBA, ICHIT (malaria)	
0.0234	20 K	Hemoglobin	
0.027	7444	Protein A-VHIII	Tumor regulation
0.031	6484	Antitumor agents (TNF + IL-2 + IFN-beta + human M-CSF)	
0.0313	6422	Oncogenes	
0.039	5154	IL-1	
0.0430	4674	Phospholipases	
0.0439	4579	Insulin multimer	
0.0446	4508	Glucocorticoide receptors	
0.0459	4379	Homeo box proteins	
0.0488	4119	Enhancers	
0.049	4102	TNF receptors	
0.051	3941	TNFs	
0.054	3722	Proto-oncogenes	
0.0590	3407	Cytochrome B	
0.062	3242	EGF–EGF receptor	
0.0703	2859	Neurotoxins	
0.0781	2574	Operators	
0.0820	2451	Interferons	
0.0820	2451	Myoglobins	
0.0839	2396	Bacterial repressors	Viral–bacterial infection
0.0947	2122	Heat shock proteins	
0.096	2094	Tubulins A + B	
0.0990	2030	Repressors	
0.1054	1907	Phage repressors	
0.110	1827	EBA-RBC (malaria interaction with red blood cells)	
0.115	1748	Myxoma virus	
0.162	1241	IGFBP	
0.173	1162	Telomere binding	
0.186	1081	HIV envelope	
0.188	1069	Telomere	
0.2363	851	Chymotrypsins	
0.281	715	Purple (bacteria)	
0.285	705	TERT + telomerase RNA + progerin	Growth
0.288	698	EGFs	
0.289	695	Growth hormons + NGF + proliferins	
0.2929	686	Growth factors (CSF + EGF + IL-2)	
0.297	678	CSF, Ubiquitins, EPA	
0.300	670	IL-2, IL-4, IL-6	
0.308	653	IL-2—IL2 receptor	
0.3203	628	Glucagons	
0.3281	613	Lysozymes	Enzymes
0.3400	591	Myosins	
0.3437	585	Promoters	
0.3447	583	Trypsins	
0.346	581	Red (rhodopsin)	
0.35	574	RNA polymerase	
0.355	566	Green (rhodopsin and chlorophylls)	
0.3555	565	Protease inhibitors	
0.3770	533	Proteases	
0.379	530	Flavodoxins	
0.3828	525	Insulin receptors	
0.383	525	Insulins	
0.4040	498	NGFs	
0.4121	488	Amylases	
0.4297	468	Kinases	
0.434	463	Tubulins beta	Structural proteins
0.4423	454	Fibrinogens	
0.449	448	Tubulins alpha	
0.4512	445	FGFs, FGF receptors	
0.453	444	IL-12	
0.4609	436	Serine proteases	
0.4687	429	SOS operators	
0.475	423	Blue (rhodopsin and bioluminescent proteins)	Blue
0.4765	422	Cytochrome C	
0.4800	419	Actins	
0.4922	408	ACH receptors	
0.4922	408	IGFs	
